# Venetoclax-Related Neutropenia in Leukemic Patients: A Comprehensive Review of the Underlying Causes, Risk Factors, and Management

**DOI:** 10.3390/ph17040484

**Published:** 2024-04-10

**Authors:** Laura Giuseppina Di Pasqua, Murwan Mahmoud Abdallah, Fausto Feletti, Mariapia Vairetti, Andrea Ferrigno

**Affiliations:** Department of Internal Medicine and Therapeutics, University of Pavia, 27100 Pavia, Italy

**Keywords:** venetoclax, neutropenia, AML, CLL, BH3 mimetic, Bcl2

## Abstract

Venetoclax is a Bcl-2 homology domain 3 (BH3) mimetic currently approved for the treatment of chronic lymphocytic leukemia (CLL) and acute myeloid leukemia (AML) that has proven to be highly effective in reinstating apoptosis in leukemic cells through the highly selective inhibition of the anti-apoptotic protein B-cell lymphoma-2 (Bcl-2). Clinically, venetoclax has provided lasting remissions through the inhibition of CLL and AML blasts. However, this activity has often come at the cost of grade III/IV neutropenia due to hematopoietic cells’ dependence on Bcl-2 for survival. As life-threatening infections are an important complication in these patients, an effective management of neutropenia is indispensable to maximize patient outcomes. While there is general consensus over dose reduction and scheduling modifications to minimize the risk of neutropenia, the impact of these modifications on survival is uncertain. Moreover, guidelines do not yet adequately account for patient-specific and disease-specific risk factors that may predict toxicity, or the role combination treatment plays in exacerbating neutropenia. The objective of this review is to discuss the venetoclax-induced mechanism of hematological toxicity, the potential predictive risk factors that affect patient vulnerability to neutropenia, and the current consensus on practices for management of neutropenia.

## 1. Introduction

While limited therapeutic options such as conventional chemotherapy and hematopoietic stem cell transplantation (HSCT) have historically rendered the treatment of hematological malignancies clinically challenging, the advent of targeted molecular therapy based on specific genetic profiles has marked a pivotal shift in the treatment paradigms with the primary aim of optimizing patient survival outcomes [1].

The global incidence of hematological malignancies is on the rise. In Europe, they constituted 8% of all new cancer cases, and 7% of cancer-related deaths in 2005 [2]. In 2021, among the nearly 2 million anticipated cancer diagnoses in the United States, 9.8% were expected to be leukemia, lymphoma, or myeloma [3]. In Australia, the incidence of blood cancers has surged by 47% in the last decade, resulting in an annual mortality rate of nearly 6000 individuals [4].

Leukemia, encompassing acute lymphocytic leukemia (ALL), chronic/small lymphocytic leukemia (CLL/SLL), acute myeloid leukemia (AML), and chronic myeloid leukemia (CML), afflicts 250,000 individuals annually. While it can manifest at any age, leukemia stands as the predominant pediatric tumor, constituting 35% of cancers in children up to the age of 14. On the other hand, most cases of leukemia manifest in individuals aged 65 and older, rendering it a malignancy affecting both the pediatric and geriatric populations [2]. Survival rates across leukemia types exhibit considerable variation, with CLL demonstrating the most favorable 5-year survival rate at 88%, while AML presents the least favorable, at 31.7% [5]. However, the individual prognosis is highly variable, with indolent and aggressive disease courses observed in CLL patients [6,7].

CLL, characterized by an accumulation of CD5+ B lymphocytes in the peripheral blood, bone marrow, and lymphoid organs due to abnormal lymphocyte production in the bone marrow, is the most prevalent leukemia in adults, constituting nearly a third of all leukemia cases [8]. Although typically diagnosed at the age of 65, a rising incidence in younger individuals has been documented: population-based studies indicate that between 7% and 20% of patients with CLL are diagnosed before the age of 55 [9,10]. CLL is considered a nonradiogenic form of cancer [11], but predisposing genetic factors have been identified [8]. CLL, which is often asymptomatic, is incidentally detected through routine blood count screenings, with less common presentations including fever, night sweats, anorexia and fatigue. Diagnosis is confirmed by the presence of “smudge cells” on peripheral blood smear and flow cytometry to ascertain clonality. Treatment has evolved from Bruton tyrosine kinase (BTK) inhibitors and alkylating agents to newer, less toxic options such as chlorambucil-obinutuzumab and combination venetoclax therapy, especially in older patients [7,8].

AML, the most common acute leukemia in adults, primarily affects individuals aged 65 and older. While it can arise secondarily to prior chemotherapy or radiation exposure, the majority of cases emerge de novo in previously healthy individuals [12]. AML’s pathogenesis involves aberrations in the maturation process of myeloid precursor cells, driven by chromosomal translocations, such as t(8;21) affecting core-binding factor or t(15;17), and genetic mutations [13]. The latter include class I mutations such as FLT3, K/NRAS [14], and TP53 aberrations [15], and class II mutations such as NPM1 and CEBPA [16]. The accumulation of malignant, poorly differentiated myeloid cells in the bone marrow and peripheral blood results in the clinical manifestations of AML: patients often present with fatigue, anorexia, and weight loss and are found to have leukocytosis, anemia, and thrombocytopenia. In some cases, AML patients may manifest symptoms related to the lack of leukocytosis and the presence of pancytopenia or other associated complications [17]. When compared to the 2016 WHO criteria that defined AML as presence of 20% blasts in the bone marrow or peripheral blood in association with any evidence of extramedullary myeloblasts or presence of AML-defining genetic abnormalities irrespective of blast percentage, the updated ICC guideline broadens the genetic abnormalities that define specific AML groups and requires at least 10% blasts in the bone marrow or peripheral blood for defining AML with recurrent genetic abnormalities (with the exception of ≥20% in AML with BCR::ABL1) [18]. Left untreated, AML is fatal within months due to infection or bleeding [12]. Standard treatment involves intensive induction chemotherapy followed by consolidation chemotherapy, often coupled with allogeneic stem cell transplantation. However, older AML individuals often face challenges in receiving standard chemotherapy due to age-related factors, necessitating less intensive treatment regimens, which may include hypomethylating agents such as azacitidine or decitabine, as well as low-dose cytarabine. In AML patients aged 65 and older, azacitidine monotherapy has been associated with remission rates of 30% or less and survival durations of under 1 year [19].

The first breakthrough in the treatment of hematological malignancies arrived in 2001 in the form of imatinib, a first-generation tyrosine kinase inhibitor (TKI) which, by targeting the ATP pocket in the BCR-ABL fusion product, blocks the constitutive activation of Abl tyrosine kinase activity, which is responsible for the uncontrolled proliferation in CML [1]. Subsequent advancement included targeted interventions addressing cancer survival mechanisms through Bcl-2 inhibition, notably exemplified by venetoclax. This BH3 mimetic has garnered approval for treatment-naïve CLL/SLL patients in combination with obinutuzumab [20] and for relapsed/refractory CLL in combination with rituximab [21]. In addition, venetoclax, in combination with azacitidine, decitabine, or low-dose cytarabine, is approved for treating AML in older adults or those with comorbidities precluding intensive chemotherapy [19,22].

Despite demonstrating promising therapeutic outcomes, these regimens present a challenge in that they tend to induce hematological toxicity, particularly in patients already predisposed to an immunocompromised state. Notably, neutropenia emerges as a predominant adverse effect of venetoclax, both in monotherapy and combination treatment [23]. Given that infectious complications constitute a significant cause of mortality and morbidity in cancer patients, particularly in the elderly and those with acute leukemia [24,25], understanding the underlying causes, risk factors, and management of neutropenia and hematological toxicity is imperative in the context of the newest mainstay treatment in hematology–oncology: venetoclax.

## 2. The Introduction of BH3 Mimetics for the Therapeutic Bcl-2 Inhibition

**Figure 1:**
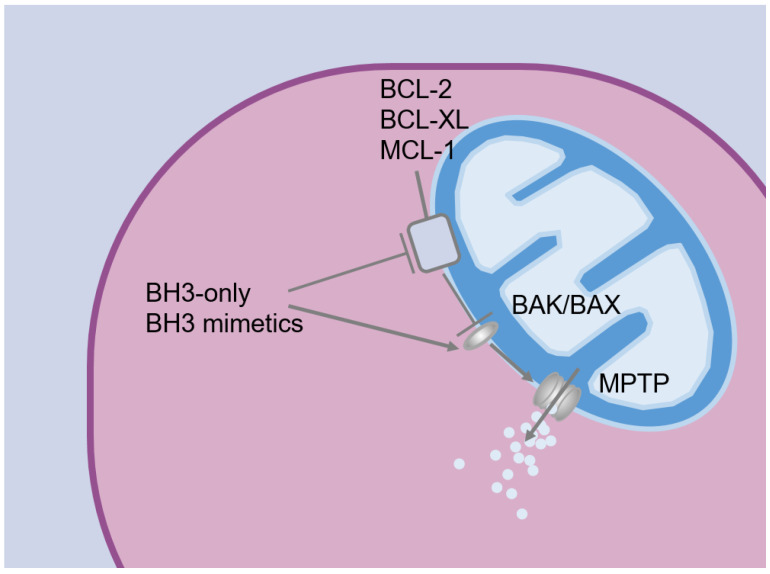
The intrinsic apoptotic pathway triggers the activation of the pro-apoptotic members of the BCL-2 protein family, known as BH3-only proteins. These BH3-only proteins interact with and neutralize pro-survival BCL-2 proteins, effectively releasing the key apoptotic effectors BAK and BAX. BAK and BAX then assemble into large complexes that induce ruptures in the mitochondrial outer membrane (mitochondrial permeability transition pore, MPTP), leading to the release of apoptogenic factors, such as cytochrome c. Notably, certain BH3-only proteins have been shown to directly bind to and activate BAK and BAX to induce MPTP as well. The BH3-only mimetic venetoclax displaces and reactivates pro-apoptotic proteins bound to the BH3-binding groove of BCL2, leading to the assembly of MPTP.

The therapeutic targeting of Bcl-2 in leukemia emerged following the elucidation of its crucial role in evading apoptosis. Bcl-2 was initially associated with a proliferative function upon its discovery in follicular lymphoma translocation (14:18), leading to its constitutive activation by enhancers of the immunoglobulin heavy chain locus [26]. It was subsequently delineated that Bcl-2 belongs to the broader BCL family of proteins, orchestrating a delicate balance between apoptosis and cell survival. Specifically, Bcl-2 assumes a prominent role in thwarting apoptosis [27]. The BCL- Homology Domains 3 (BH3-Only) proteins coordinate cell death signaling by directly activating apoptotic effectors BAX and BAK, and concurrently suppressing anti-apoptotic proteins. The branch of the BCL family that includes Bcl-2, BCL-XL, and MCL-1, counteracts the propagation of cell death signals by binding BH3-only proteins (Figure 1) [28,29,30,31]. Consequently, BCL2 assumes a critical anti-apoptotic function by binding and sequestering pro-apoptotic proteins, thereby precluding their interaction with apoptotic effectors BAX and BAK. Consequently, mitochondrial outer membrane permeabilization, requisite for the release of cytochrome c and the activation of the final caspase-dependent cell death sequence, is impeded [32].

Both healthy and malignant cells rely on the aforementioned anti-apoptotic proteins for survival. Notably, various hematological malignancies exhibit Bcl-2 overexpression, with specific malignancies exploiting this pro-survival mechanism for unrestricted growth. In particular, Bcl-2 plays a direct role in tumorigenesis and resistance to chemotherapy. In CLL, for instance, nearly every patient demonstrates Bcl-2 overexpression; a consequence of genomic deletion (13q14) impairing the microRNA inhibitors of Bcl-2 (miR15 and miR16) in half of these patients. Similarly, Bcl-2 overexpression is universally found in SLL, frequent in Mantle Cell Lymphoma and Waldenstrom Macroglobulinemia, and expressed in specific subsets of other leukemias and lymphomas [33,34].

The first attempt at blocking Bcl-2 for treatment involved the antisense oligodeoxyribonucleotide oblimersen and Bcl-2 inhibitor obatoclax. However, Phase I/II trials showed negligible activity in monotherapy as a consequence of a suboptimal ability to bind Bcl-2 [35]. Obatoclax was similarly ineffective but also showed dose-limiting neurotoxicity [36]. Promising anti-Bcl-2 activity was finally achieved with navitoclax, a modified form of obatoclax designed to optimize the interaction with Bcl-2 through high-specificity binding. However, in a Phase I study (Study M06-873), navitoclax exhibited the opposite problem of its predecessor in that it bound BCL-XL with such a high affinity that it adversely affected platelet count, leading to acute severe thrombocytopenia [37]. This hematological toxicity is a direct consequence of platelet dependence on BCL-XL for survival [38]. Thus, while demonstrating promising Bcl-2-specific activity against chronic lymphocytic leukemia, Study M06-873 also established that the therapeutical efficacy of navitoclax dose scheduling and escalation is impaired by the need to interrupt or discontinue therapy in a number of patients due to the development of grade 4 thrombocytopenia [37].

In the pursuit of maximizing therapeutic benefits while minimizing toxicity, navitoclax was further refined to produce the potent Bcl-2 inhibitor venetoclax. Venetoclax has become the only selective BH3 mimetic currently approved by the United States Food and Drug Administration (FDA) for the treatment of CLL/SLL in adult patients and, in combination with azacitidine, decitabine, or low-dose cytarabine, for newly diagnosed AML in adults 75 years or older, or who have comorbidities precluding intensive induction chemotherapy [39,40].

**Figure 2:**
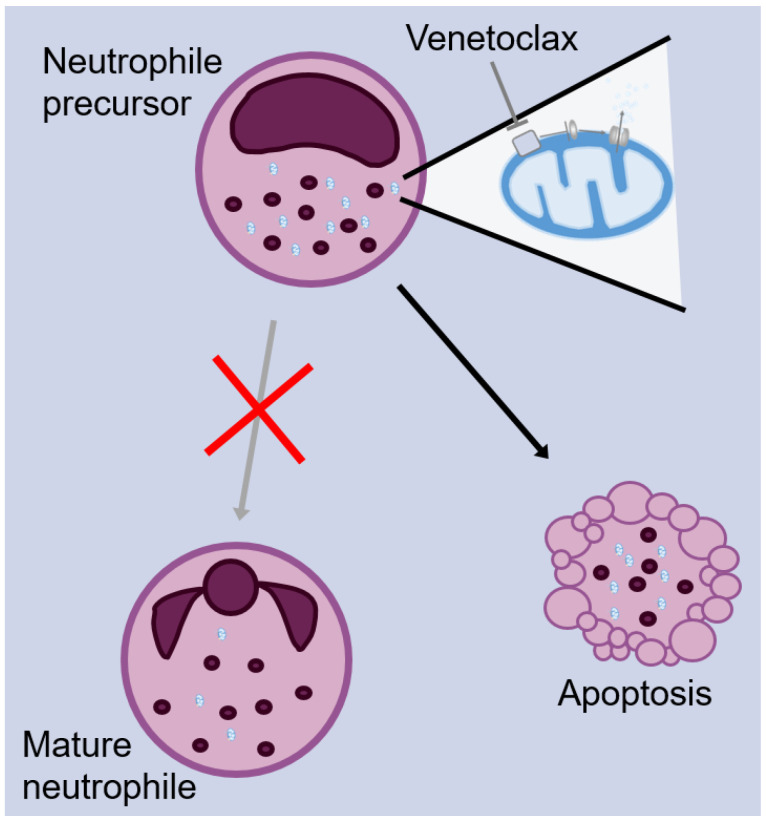
Mechanism of venetoclax-induced neutropenia. The anti-apoptotic Bcl-2 expression is readily detectable at the protein level on neutrophil precursors, while it is very low in mature neutrophils. Bcl-2 inhibition triggers apoptosis in neutrophile precursors, resulting in neutropenia.

Venetoclax is distinguished from its predecessor in that, while it binds the Bcl-2 binding domain with high specificity, it also exhibits high selectivity for Bcl-2 over BCL-XL, preserving the anti-apoptotic activity critical for platelet survival. In fact, its Bcl-2 inhibition potency surpasses that of navitoclax while demonstrating 200 times less activity against BCL-XL, ensuring lethal activity against malignancy while preventing potential dose-limiting toxicity. Furthermore, it does not inhibit MCL-1, another anti-apoptotic BCL family member on which healthy hematopoietic cells primarily rely for survival [41]. The neutrophil precursors, however, are dependent upon Bcl-2 for survival, and this dependency is proposed to underlie venetoclax’s most common adverse effect, neutropenia (Figure 2) [34].

## 3. Efficacy and Hematological Toxicity in Clinical Trials

### 3.1. Efficacy in Chronic Lymphocytic Leukemia

The groundbreaking phase I clinical trial evaluating venetoclax efficacy in leukemia was conducted by Roberts et al., assessing venetoclax monotherapy for relapsed/refractory (R/R) CLL. Fifty-six patients with R/R CLL were enrolled and divided into 8 venetoclax dose-escalation groups ranging from 150 to 1200 mg per day, with the peak target dose achieved by week 3. Venetoclax demonstrated efficacy at all dosages, inducing significant reductions in the CLL cell burden. However, due to the detection of tumor lysis syndrome (TLS) in three patients, including one TLS-associated fatality, a dosage ramp-up schedule from 20 mg/day to a maximum of 400 mg/day over five weeks was implemented in an expansion cohort of 60 patients. Among the total of 116 patients enrolled in this trial, 92 responded to venetoclax (an overall response rate of 79%), with 20% achieving complete remission. Sixty-nine percent of the 400 mg expansion cohort attained 15-month progression-free survival, and the 2-year overall survival for all patients was 84%. Notably, this cohort included patients with high-risk factors such as multiple prior therapies, TP53 aberrations, and unmutated immunoglobulin heavy variable (IGHV) genes. Venetoclax achieved encouraging response and remission rates in these high-risk subsets as well, further highlighting its therapeutic potential. Neutropenia was another prominent adverse effect, with 41% of patients experiencing grade 3 or 4 neutropenia during the study [42].

Building upon prior clinical evidence of synergistic action between navitoclax and the anti-CD20 monoclonal antibody rituximab [43], Seymour et al. undertook a Phase 1b study to evaluate the efficacy of venetoclax in combination with rituximab for the treatment of R/R CLL. Forty-nine patients were enrolled and received venetoclax in escalating doses to a target range of 200–600 mg, followed by monthly rituximab administration (375 mg/m^2^ in the first month and 500 mg/m^2^ in subsequent months). The results were once again encouraging, with 51% of patients achieving a complete response and 57% exhibiting undetectable measurable residual disease (u-MRD). Two-year progression-free survival was estimated to be 82%. The most common grade 3/4 adverse event was neutropenia (53%), followed by thrombocytopenia (16%), anaemia (14%), febrile neutropenia (12%), and leucopenia (12%); however, an increase of severe infections was not observed [44]. The most recent follow-up of this study yielded a 5-year progression-free survival estimate of 56% and an overall survival estimate of 86%. Notably, there were no instances of febrile neutropenia reported after the first 2 years of treatment [45].

The phase II study conducted by Stilgenbauer et al. represented a significant turning point for venetoclax, paving the way for its FDA approval in the treatment of R/R CLL/SLL with 17p deletion. Prior to venetoclax, it was widely recognized that the deletion of chromosome 17p in CLL patients conferred a poor prognosis, characterized by inadequate response to standard chemo-immunotherapy, short progression-free survival, and reduced overall survival [46]. While B-cell receptor signaling inhibitors (BCRi) such as ibrutinib and idelalisib were more promising than conventional therapy in phase I/II trials, treatment was often hindered by interruptions or discontinuations due to toxicity concerns, drug–drug interactions, and disease progression [47,48]. This prompted Stilgenbauer et al. to investigate venetoclax monotherapy as a therapeutic option in a phase II study, enrolling 107 R/R CLL patients with 17p deletion to receive a target dose of 400 mg following a five-week ramp-up period. The overall response rate was 79.4%, with 69% of patients achieving partial remission and 8% attaining complete remission [38]. An additional 51 patients were enrolled as a safety expansion cohort, bringing the total enrollment to 158 patients. The phase II study demonstrated that 2-year progression-free survival and overall survival were 54% and 73%, with 30% of patients achieving undetectable minimal residual disease. Grade 3/4 AEs were primarily hematologic, including neutropenia, thrombocytopenia, and anemia (40%, 15% and 15%, respectively). Pneumonia was the most common serious AE (10%). Venetoclax dosing was reduced in twenty-seven patients (17%), and interrupted in 63 (40%) as a result of AEs, with neutropenia being the most common reason for dose adjustments [49,50].

These early clinical trials showed that venetoclax could cause deep and lasting remissions in many patients, even those with high-risk genetic features. Also, the fact that most patients in remission had undetectable measurable disease after venetoclax treatment led to the use of fixed-duration cycles of venetoclax in later trials. Finally, these early studies confirmed that the most frequent grade 3/4 adverse effects were of hematological origin.

MURANO, the first phase III trial with venetoclax, compared the combination of venetoclax and rituximab with standard chemoimmunotherapy (bendamustine plus rituximab) in relapsed/refractory CLL patients. The study randomly assigned 389 patients to either receive venetoclax plus rituximab, or bendamustine plus rituximab for 6 months. After a median follow-up of 23.8 months, progression-free survival was found to be much longer in the venetoclax-rituximab group (89.4%) versus the bendamustine-rituximab group (36.3%). In addition, the overall response rate was higher at 92.3% for the former group than the 72.3% of the latter. Prominently, this efficacy was consistent regardless of 17p deletion status; the 2-year progression-free survival rate was higher in the venetoclax–rituximab group (81.5%) than the bendamustine–rituximab group (27.8%) among patients with 17p deletion, but was also higher among patients without it (85.9% in the venetoclax–rituximab group versus 41% in the bendamustine–rituximab group). Moreover, among the 26.3% of patients who had TP53 mutations and 68.3% with unmutated IGHV status, the benefit of the venetoclax–rituximab regimen was consistent [21]. These findings became the basis for FDA approval of venetoclax for the treatment of relapsed/refractory CLL/SLL, regardless of 17p deletion status. Three-year progression-free survival remained significantly higher with venetoclax–rituximab (71.4%) compared to bendamustine–rituximab (15.2%) at 36 months follow-up [51]. At the 4-year follow-up, long-term outcomes were reported, particularly in patients who achieved uMRD; a good response was also observed for salvage therapy with ibrutinib [52].

Venetoclax also emerged as a promising therapy for treatment-naïve patients in the 2019 CLL14 trial by Fischer et al. This randomized phase III trial enrolled 432 untreated CLL patients and randomly assigned them to either a fixed-duration combination of venetoclax and obinutuzumab or chlorambucil and obinutuzumab, each lasting 12 cycles of 28 days. Both groups received obinutuzumab for the first six cycles at 1000 mg. In the venetoclax–obinutuzumab arm, venetoclax was introduced on day 22 of cycle 1, gradually increased to 400 mg over five weeks, and maintained at 400 mg daily until the end of cycle 12. Compared to the chlorambucil–obinutuzumab group, the venetoclax–obinutuzumab group achieved significantly higher rates of complete response and minimal residual disease negativity in both peripheral blood (42.1% vs. 14.4%) and bone marrow (33.8% vs. 10.6%). Additionally, the venetoclax–obinutuzumab arm demonstrated superior progression-free survival at 24 months regardless of mutation status, reinforcing its efficacy in high-risk subsets as observed in the MURANO trial. Overall, the venetoclax–obinutuzumab group exhibited a significantly higher 24-month progression-free survival rate (88.2% vs. 64.1%) [20]. Adverse events associated with grade III/IV neutropenia observed during the clinical trials of venetoclax for CLL/SLL are outlined in Table 1.

### 3.2. Efficacy in Acute Myelogenous Leukemia

The promising clinical activity of venetoclax in treating CLL encouraged research of its potential in myeloid malignancies. The first phase II trial by Konopleva et al. evaluated venetoclax alone in relapsed or refractory AML and AML unfit for intensive therapy. To avoid tumor lysis syndrome seen in CLL trials, a ramp-up schedule to the target dose over 6 days was adopted. The dosing regimen started at 20 mg on day one and reached 800 mg daily on day 6. After the first assessment, in case of incomplete remission or complete remission with incomplete hematological recovery, the dose was increased to 1200 mg daily. A 19% overall response rate was observed, with 6% complete response and 13% complete remission with incomplete hematological recovery. These results showed modest venetoclax efficacy, but Bcl-2 family protein analysis and BH3 profiling showed that AML blast Bcl-2 upregulation and dependence varied in these patients, suggesting that venetoclax could work better with a combination regimen [60].

The phase 1b dose-escalation study by DiNardo et al. was the first trial to evaluate combination treatment with venetoclax and a hypomethylating agent in AML patients. In this study, venetoclax was administered in conjunction with either decitabine or azacitidine to treatment-naïve patients aged over 65 who were not eligible for intensive chemotherapy. Patients received venetoclax following a short ramp-up dosing schedule (3 to 5 days) reaching a target dose of 400, 800, or 1200 mg daily. Decitabine was administered for the first 5 days of each cycle (20 mg/m^2^), while azacitidine was administered for the first 7 days of each cycle (75 mg/m^2^). Remarkably, 67% of patients across all dose levels achieved complete remission, with a median overall survival of 17.5 months. Notably, half of the study participants had poor-risk cytogenetics (TP53, FLT3, IDH1/2, MPM1 aberrations) [61].

Further corroborating this finding, a phase Ib/II trial conducted by Wei et al. investigated the efficacy of venetoclax combined with low-dose cytarabine (LDAC) in treatment-naïve AML patients aged 60 years and older. Venetoclax was gradually escalated to a target dose of 600 mg or 800 mg over a period of 4 to 5 days, with subsequent 28-day cycles commencing at the target dose. LDAC was administered on the first 10 days of each cycle (20 mg/m^2^). This regimen yielded an overall complete response rate of 54%, with a median duration of remission reaching 8.1 months. Notably, the highest complete response rates were observed in treatment-naïve patients with de novo AML (71%), intermediate-risk cytogenetics (63%), and no prior exposure to hypomethylating agents (62%) [62].

The findings of these trials led to the accelerated FDA approval of venetoclax in combination with hypomethylating agents or LDAC as a frontline therapy for patients aged 75 and older who are not eligible for intensive chemotherapy. However, full approval came after the pivotal phase III VIALE-A and VIALE-C trials, which explored the effectiveness of venetoclax combinations in patients with comorbidities that ruled out intensive chemotherapy.

The VIALE-C trial evaluated the efficacy of venetoclax in combination with LDAC compared to LDAC alone in newly diagnosed AML patients aged 18 years or older who were ineligible for intensive chemotherapy. Participants were randomly assigned to receive either venetoclax plus LDAC or placebo plus LDAC in both groups. Venetoclax dosing was initiated at 100 mg on day 1 and gradually escalated to a target dose of 600 mg by day 4, which was maintained for subsequent 28-day cycles. Both treatment arms received LDAC at a dose of 20 mg/m^2^ administered on the first 10 days of each cycle. The trial demonstrated a remarkable difference in complete remission rates between the two groups, with 48% of venetoclax-LDAC patients achieving complete remission compared to only 13% in the LDAC alone group. This compelling efficacy advantage extended to overall survival, that, in an unplanned 6-month follow-up, showed a median value of 8.4 months in the venetoclax–LDAC group compared to 4.1 months in the LDAC alone group [22,63].

The VIALE-A study enlisted previously untreated patients aged 18 and above, either ineligible for standard chemotherapy or aged 75 or older (or both). They were allocated to receive either venetoclax plus azacitidine or azacitidine plus a placebo as a control. As in earlier trials, venetoclax was gradually increased to 400 mg daily in 28-day cycles. Both the venetoclax and placebo groups were administered 75 mg/m^2^ of azacitidine for the initial 7 days of each cycle. Complete remission was achieved by 66.4% of patients in the venetoclax–azacitidine group compared to 28.3% in the control group. Importantly, with a median follow-up of 20.5 months, the overall survival reached 14.7 months in the venetoclax–azacitidine treatment group and 9.6 months in the control cohort [19].

AML is primarily a disease of the elderly with a median reported age at diagnosis of around 70 years. In contrast to younger patients, whose 5-year overall survival rates improved significantly since the 1970s, survival in elderly patients remained poor [64]. Recently, the combination of venetoclax and hypomethylating agents was shown to strongly improve response and survival in frail elderly AML patients [19]; however, the clinical course is sometimes still characterized by frequent septic complications [65]. Adverse events associated with grade III/IV neutropenia observed during the clinical trials of venetoclax for AML are outlined in Table 2.

## 4. Hematological Toxicity

Clinically, it has been observed that neutropenia is the most common adverse effect of venetoclax, both in monotherapy and combination treatments [67]. Neutropenia is identified through a complete blood count, and its severity is categorized based on the absolute neutrophil count (ANC), which typically ranges from 1500 to 8000 cells/µL of blood in adults. Neutropenia is defined as an ANC < 1500 cells/mm^3^ and is further stratified into grades in accordance with National Cancer Institute Common Toxicity Criteria (NCI-CTC) [68]. Grade I occurs when ANC is at least 1500 but less than 2000 cells/mm^3^, grade II between 1000 and 1500 cells/mm^3^, grade III between 500 and 1000 cells/mm^3^, and grade IV when it falls below 500 cells/mm^3^. This grading is clinically significant due to the escalating risk of infection with the progressive decline of ANC, leading to a compromised immune system defense against endogenous mucosal gastrointestinal (GI) and mucosal flora, as well as opportunistic fungal infections. Grade IV neutropenia, in particular, poses a severe risk of life-threatening infections and is a significant predictor of dose reductions, interruptions, and impaired relative dose intensity [68,69].

In laboratory settings, Bcl-2 inhibition with venetoclax has been shown to induce neutropenia in rats and inhibit granulocyte colony formation in human bone marrow samples [28]. The mechanism of neutropenia induction has not been fully elucidated but is attributed to venetoclax-induced selective killing of granulocyte progenitors [70].

The aforementioned clinical trials have consistently highlighted grade III/IV neutropenia and febrile neutropenia as predominant hematological adverse effects in the treatment of both CLL and AML with venetoclax. In the phase I venetoclax monotherapy trial for CLL, conducted by Roberts et al. (2016), neutropenia emerged as the most common grade III/IV adverse event, affecting 41% of patients with 6% experiencing grade IV neutropenia. The leading serious adverse event was febrile neutropenia, impacting 6% of patients [42]. In the subsequent phase 1b trial led by Seymour et al. (2017), grade III/IV neutropenia was observed in 53% of patients, febrile neutropenia in 10%, thrombocytopenia in 16%, and anemia in 14% of patients [44].

In the phase II trial of venetoclax monotherapy by Stilgenbauer et al. (2018), hematological toxicity was prevalent, with 40% of patients experiencing grade III/IV neutropenia, 15% facing thrombocytopenia, and 25% encountering anemia. The infection rate reached 81%, including pneumonia in 10% of patients. Consequently, 17% of patients necessitated venetoclax dose reduction, and 40% experienced dosing interruptions [49]. Additionally, in the pivotal Phase III MURANO trial, neutropenia emerged as the most common adverse event of any grade and the most frequent grade III/IV adverse event. It was reported in 58% of patients in the venetoclax–rituximab treatment group compared to 70.2% in the bendamustine–rituximab cohort [21].

Notably, treatment with monoclonal antibodies has been identified as an independent factor contributing to neutropenia development. Therefore, combination treatments should be regarded as an exacerbating factor in increasing the occurrence of neutropenia [71].

In the first controlled trial evaluating venetoclax efficacy in AML patients, the most common grade III/IV adverse event was febrile neutropenia, occurring in 31% of patients. This incidence was consistent with expectations for this patient population, given the median age of 71 years [60]. Consistent with these safety findings, the phase 1b trial of venetoclax in combination with hypomethylating agents identified febrile neutropenia as the most prevalent grade III/IV adverse event, affecting 43% of patients. Leukopenia (31%), anemia (25%), thrombocytopenia (24%), and neutropenia (17%) were also observed at increased rates [60]. The phase Ib/II trial by Wei et al. demonstrated that patients receiving 600 mg of venetoclax were able to maintain treatment without dose interruptions, while those in the 800 mg cohort frequently required dose reductions due to severe cytopenia. Based on these findings, the recommended dose of venetoclax in combination with LDAC was established at 600 mg [62]. Febrile neutropenia (42%), thrombocytopenia (38%), neutropenia (27%), and anemia (27%) were the most common grade III/IV adverse events in this trial [62].

The VIALE-A and C studies also revealed a higher incidence of hematological toxicity, particularly neutropenia and febrile neutropenia, in the treatment regimen that incorporated venetoclax. In the VIALE-A trial, while a comparable number of patients experienced anemia and thrombocytopenia, grade III/IV neutropenia occurred in 42% of the venetoclax–azacitidine arm compared to 28% in the control arm, and grade III/IV febrile neutropenia occurred in 42% and 19%, respectively [19]. Additionally, although the proportion of patients who discontinued treatment due to adverse effects was similar in both groups, dose interruptions—principally due to neutropenia and febrile neutropenia—occurred more frequently in the venetoclax–azacitidine cohort (72%) than the control group (57%) [19]. Elevated rates of neutropenia (47% vs. 16%), febrile neutropenia (32% vs. 29%), and thrombocytopenia (45% vs. 37%) were also observed in the venetoclax–LDAC arm compared to the control group in the VIALE-C study [22].

A recent assessment of trial data from the VIALE-A study clarified that venetoclax-induced neutropenia is not confined to patients receiving active treatment; it was also observed in patients in remission. The post hoc analysis evaluated the incidence of cytopenia in VIALE-A patients who had achieved a complete response or complete response with partial hematologic recovery to therapy. Among these patients, post-remission grade IV cytopenia persisting for at least 7 days occurred in 87% of the venetoclax–azacitidine treatment group compared to 45% of the placebo–azacitidine cohort. Additionally, 78% of patients in the venetoclax treatment group experienced post-remission treatment cycle delays due to cytopenia, compared to 33% in the placebo–azacitidine arm. Another observation pointing to the venetoclax post-remission toxicity was that, among these patients, 74% required a reduction in venetoclax dosing days with or without delayed treatment cycles, compared to 27% in the placebo group [72].

## 5. Management of Venetoclax-Induced Neutropenia

Myelosuppression, primarily manifested as treatment-induced neutropenia with venetoclax, represents a significant dose-limiting toxicity due to the associated risk of infection and development of life-threatening febrile neutropenia. In the context of treating hematological malignancies, the challenge of maintaining relative dose intensity (RDI) while offsetting the occurrence of neutropenia and febrile neutropenia is crucial to achieve optimal clinical outcomes. Given the proven efficacy of venetoclax despite its tendency to induce neutropenia, it is imperative to identify patient subgroups for whom hematological toxicity poses the greatest risk, establish treatment regimens that optimize the benefit–toxicity ratio, and proactively mitigate the need for dosage reduction and interruption [73]. Tailoring treatment plans involving venetoclax to individual patients and disease-specific characteristics that predispose to increased risk of neutropenia and febrile neutropenia is paramount. This necessitates a comprehensive understanding of clinical prognostic factors, encompassing patient-specific risk factors and biochemical indicators, to formulate a well-tolerated and efficacious treatment strategy [71]. Notably, specific risk factors for neutropenia in the context of malignancy, such as advanced age, prior exposure to radiation or chemotherapy, comorbidities, pre-existing neutropenia, and malignancy with bone marrow involvement, should be carefully considered. For instance, the treatment of elderly patients, aged 65 and above, requires meticulous planning due to the significantly heightened risk of morbidity and mortality associated with treatment-induced hematological toxicity [74]. The approval of venetoclax for use in combination with chemotherapies in the treatment of acute myeloid leukemia (AML) in the United States was expedited due to the historical detrimental toxicity and inferior clinical outcomes associated with intensive induction chemotherapy in elderly patients. Additionally, pre-treatment assessment of other patient-specific risk factors, including comorbidity indices related to liver, kidney, and cardiovascular health, but especially performance status, particularly indicated by an Eastern Cooperative Oncology Group (ECOG) performance score > 3, is crucial in predicting treatment toxicity and prognosis. The multifaceted nature of treating elderly leukemia patients, characterized by diminished performance status, increased comorbidities, elevated risk of adverse events, pre-existing hematological disease, and polypharmacy, underscores the exceptional complexity of their management [75,76]. Biochemical indicators are also vitally important as, in addition to establishing and tracking important parameters like absolute lymphocyte count, serum markers such as lactate dehydrogenase can serve as a valuable tool in gauging a prognostic outlook before and during the course of treatment [67].

Cytogenetic analysis of disease-specific mutations can help evaluate the potential benefits of a venetoclax-inclusive regimen. For example, studies have identified cytogenetic signatures such as TP53, FLT3, IDH1/2, and NPM1 mutations, as well as chromosomal deletions like 17p, which are classified as adverse-risk. These have historically required prolonged or higher-dose treatment, increasing the risk of neutropenia. Additionally, subsets of leukemic cells with distinct transcription profiles characterized by the upregulation of BCL-2 family anti-apoptotic proteins such as BCL-XL and MCL-1, have been identified, leading to leukemic cell survival. These findings suggest that not all patients approved for venetoclax would benefit from its administration [75,77]. Additionally, leukemias with biallelic p53 mutations were found to benefit the least from venetoclax in the VIALE-A and VIALE-C trials. This is due to potentially increased dependence on BCL-XL and the mitochondria-stabilizing chaperonin CLPB for survival [78]. Venetoclax resistance mechanisms are not intrinsic to treatment-naïve patients. Some patients, after an average prolonged exposure of 36 months to the BH3-mimetic, exhibited a Gly101Val mutation that impaired the drug’s binding. Others gained resistance through the upregulation of MCL-1 and BCL-XL [79,80]. This finding has led to the suggestion that the most effective use of venetoclax in combination therapy should be on a time-limited basis to minimize toxicities like neutropenia and avoid the evolution of resistance strategies by the target malignancy. Recent evidence suggests that resistance mutations to venetoclax have only emerged after prolonged exposure, while patients treated for shorter periods retained sensitivity to Bcl-2 inhibition when disease relapse occurred. Therefore, part of minimizing venetoclax toxicity involves identifying the subset of patients in which its use, although approved and indicated, is actually ineffective and unnecessary. Molecular assays can be used to detect these resistance mechanisms [80].

Neutropenia incidence is greater in combination therapy than in venetoclax monotherapy, making chemotherapy intensity a crucial factor. The type, dose, and number of cytotoxic agents in a treatment regimen significantly impact hematotoxic myelosuppression risk. The VIALE-A trial demonstrates the significance of chemotherapy intensity, as it involved reducing the dose of azacitidine, the companion chemotherapy to venetoclax, if a 25% improvement in ANC and platelet count was not observed in cytopenic patients within 14 days of cycle completion [19]. Moreover, a recent phase II study combining venetoclax with frontline bendamustine and obinutuzumab to treat follicular lymphoma attained a complete response rate of 73%, but also resulted in a 56% incidence of serious adverse effects, specifically opportunistic infections due to myelosuppression [81]. Another example is the BELLINI trial, a phase III study combining venetoclax with bortezomib and dexamethasone in the treatment of multiple myeloma. In this study, the addition of venetoclax significantly increased response rates and progression-free survival; however, this was associated with eight fatal infections in that cohort [82]. Thus, a viable approach to minimizing venetoclax cytotoxicity involves both careful selection and the dosage modulation of combination chemotherapies because, while combination therapy may offer a synergistic therapeutic benefit, it also compounds the risk and severity of toxicity.

The response to treatment and the risk of developing hematological toxicity depend significantly on genetic variation, affecting both the tolerance of healthy hematopoietic cells to treatment and the efficacy against the genetic arsenal of the malignancy. In the absence of pharmacogenetic testing, the first cycle of cancer therapy serves as a crucial predictive and prognostic tool. Typically initiated at full dose, the first cycle poses the highest risk of neutropenia, febrile neutropenia, and infection. Subsequent cycles often see a decrease in neutropenic events due to responsive, adaptive treatment adjustments, such as prophylactic measures like antibiotics and G-CSF, as well as dose delays and reductions when necessary [69,83].

In order to minimize the risk of first cycle toxicity, in particular TLS manifestation, all current dosing regimens for CLL/AML include a ramp-up period to reach the target dose. When using venetoclax to treat CLL/SLL, the dosing is increased over a 5-week period, starting at 20 mg in the first week and reaching 400 mg in the fifth week. This escalation of venetoclax dosage can be synchronized with combination treatments, either before or after. For example, in the MURANO phase III trial, venetoclax was escalated to the target dose of 400 mg over five weeks before starting rituximab [52]. In contrast, the CLL14 trial initiated treatment with the monoclonal antibody obinutuzumab before starting the venetoclax ramp-up. This approach allowed for the normalization of patients’ absolute neutrophil count during the interim period [58].

The foundation for managing venetoclax-induced neutropenia in the treatment of CLL was established by the protocols in the AbbVie trials. These protocols set the precedent for managing neutropenia based on its frequency and severity. In cases of grade 3 neutropenia with infection/fever or grade 4 neutropenia, venetoclax administration should be interrupted until neutropenia is reduced to grade 1 or ANC returns to the baseline level. The dosage at the resumption of venetoclax depends on whether the patient is experiencing new-onset neutropenia. If it is the first occurrence, venetoclax therapy resumes at the last dose reached during the ramp-up. If it is a recurrence, the dose is reduced to 300, 200, 100, 50, or 10 mg for a pre-interruption dose of 400, 300, 200, 100, 50, or 20 mg, respectively [34,44,49]. Although studies investigating the impact of venetoclax dose delays, interruptions, and modifications have on survival outcomes are ongoing, such treatment changes have historically been associated with increased morbidity and reduced survival outcomes [84]. Trials have shown that granulocyte colony-stimulating factor (G-CSF) has the potential to reduce the duration of neutropenia, thereby preserving the therapeutic benefit of venetoclax by avoiding premature discontinuation. Despite higher rates of neutropenia, the infrequent discontinuation of doses in trials combining venetoclax with monoclonal antibody therapy can be attributed to the administration of G-CSF to nearly half of the patients in these studies. The study protocol mandated the administration of G-CSF for neutropenia, as indicated, and the provision of prophylactic G-CSF in subsequent cycles [34,67]. Future data may shed light on whether these treatment adjustments in favor of alleviating neutropenia are consequently adversely affecting survival.

In the case of AML, the rapid disease course necessitates a similarly rapid venetoclax dose escalation over 3 days, to avoid the risk of TLS [42], with the target dose set at 400 mg when venetoclax is used in combination with hyper-methylating agents, and 600 mg when used with LDAC. The VIALE-A trial demonstrated that dose delays and modifications in response to neutropenia should not occur until complete remission is achieved. Therefore, management of neutropenia in AML should be guided by bone marrow assessment for remission status. Until remission is reached, the treatment protocol should not be compromised. Instead, neutropenia should be addressed with prophylactic antimicrobial agents and supportive blood products. In the post-remission period, treatment can be interrupted if marrow assessment determines a blast count below 5%, in which case treatment is only resumed when the ANC rises above 500 cells/µL or is accelerated by G-CSF administration. Moreover, the development of post-remission grade 4 neutropenia warrants treatment delay until the recovery of the ANC above 500 cells/µL. If the AML patient experiences prolonged or new onset neutropenia despite interventions such as G-CSF, antimicrobials, and supportive care, then a bone marrow biopsy to assess treatment response is warranted. In case of second or subsequent recurrences of grade 4 neutropenia after AML remission, the measures to be applied are similar to the abovementioned, accompanied by a 7-day reduction in the duration of each subsequent venetoclax cycle (e.g., a 21-day cycle instead of a 28-day cycle) [67]. Notably, the VIALE-A trial protocol both delayed the resumption of venetoclax–azacitidine treatment and also reduced the number of days of administration when patients experienced thrombocytopenia in addition to neutropenia. In such cases, continued treatment was contingent upon both ANC recovery and a platelet count > 50 × 10^3^/µL [19,72]. Filgrastim is the recombinant G-CSF administered to combat the depletion of bone marrow granulocytes by cancer therapy. It replenishes the neutrophil count by stimulating granulopoiesis, accelerating progenitor cell proliferation and differentiation. This is an important concurrent treatment, as it has been demonstrated to reduce the risk of severe neutropenia, febrile neutropenia, and life-threatening infections due to chemotherapy. A phase Ib/II study by DiNardo et al. (2021) was conducted to assess combination therapy with fludarabine, cytarabine, G-CSF, idarubicin, and venetoclax (FLAG-IDA + VEN) in younger AML patients, with a median age of 44 years. The study included the administration of filgrastim during both the induction and consolidation phases. Despite the inclusion of venetoclax, the frequency of adverse events, such as febrile neutropenia (44%), pneumonia (24%), and bacteremia (22%), was consistent with the current standard induction chemotherapy [85]. Emerging clinical experience supports the use of filgrastim to optimize venetoclax treatment while minimizing toxicity in the management of AML. Maiti et al. (2022) reported that growth factors were effectively used to reduce the duration of neutropenia. For patients who achieved remission or hypocellular marrow on cycle 1 day 21, or day 14 with FLT3 inhibitor triplet, daily filgrastim was used until ANC trends were greater than 1.5 × 10^9^/L. Similarly, daily filgrastim was used for patients presenting with infectious complications [86].

## 6. Pharmacokinetics and Drug-Drug Interactions

A thorough understanding of venetoclax pharmacokinetics is paramount to optimize its clearance, as an increase in venetoclax bioavailability can lead to a higher risk of adverse events, including neutropenia.

Venetoclax has an oral bioavailability of 5.4% [87]. It reaches peak concentration approximately five to eight hours post-intake, with a mean terminal half-life of 14–18 h [88]. The bioavailability of venetoclax is significantly influenced by food intake; when taken with a high-fat meal, the C_max_ and AUC_0–∞_ of venetoclax increased by 3.68-fold and 4.42-fold, respectively. Similarly, when given after a low-fat meal, the AUC_0–24_ of venetoclax increased by 4.27-fold compared to the value observed under fasting conditions [87]. This is likely due to the increased intestinal lymphatic transport of the drug and absorption into systemic circulation, which bypasses hepatic first-pass metabolism when food is consumed [89]. Therefore, it is recommended that venetoclax be taken with a meal.

Venetoclax is highly protein-bound in plasma (>99%) and has a large volume of distribution at steady state. In male and female cancer patients, the volume of distribution is approximately 321 L and 256 L, respectively [89]. The systemic clearance of venetoclax occurs primarily through hepatic elimination, with metabolism being predominantly handled by CYP3A family enzymes. Total clearance of the drug from plasma after oral administration decreases by 19% in the case of moderate CYP3A inhibitors, and it can further decrease by 84% in the case of potent CYP3A inhibitors [90]. Renal clearance is negligible, accounting for less than 1% of the total clearance; in fact, mild and moderate renal impairment were not found to impact venetoclax exposures in a population pharmacokinetic analysis of eight clinical studies [90]. Moreover, mild-to-moderate hepatic disease does not significantly affect venetoclax clearance. No data are available in subjects with severe hepatic or renal impairment, so further studies may be required given venetoclax CYP3A metabolism [90].

Since venetoclax is metabolized by CYP3A family enzymes, its concomitant administration with other drugs processed by this system can lead to reduced clearance and increased venetoclax levels, raising the risk of adverse events such as neutropenia. One such example is the antifungal azole group, often given preventively for treatment-induced myelosuppression. A study by Agarwal et al. (2017) examined the impact of venetoclax combined with posaconazole in AML patients. It was found that the co-administration of posaconazole increased the plasma concentration of venetoclax by 53% and 93% at doses of 50 mg and 100 mg, respectively; additionally, its AUC_0–24_ increased by 93% and 155% at the same doses [91]. Current recommendation dictates that strong CYP3A inhibitors should not be used during the five-week ramp-up in CLL/SLL treatment. If necessary, these should be administered after the ramp up, with a 75% dose reduction for venetoclax [92,93]. Significantly, in contrast to CLL/SLL patients, AML patients are permitted to receive CYP3Ai antimicrobial prophylaxis during the venetoclax ramp-up due to the augmented risk of severe neutropenia originating from the short, aggressive ramp-up schedule. In such cases, the venetoclax dose is to be capped at a maximum of 100 mg [23,67]. Weak inhibitors for CYP3A also have significant drug interactions with venetoclax. In this category, the following are included: azole antifungals such as fluconazole and isavuconazole; protease inhibitors such as amprenavir, atazanavir, darunavir/ritonavir; calcium-channel blockers such as: diltiazem, verapamil; and antimicrobial drugs such as ciprofloxacin and erythromycin. When used in combination with weak CYP3A inhibitors, it is currently recommended to reduce the dose of venetoclax by 50%, and closely monitor any potential adverse drug reactions [94].

Venetoclax is also a substrate and inhibitor of ABC-family transporters P-gp and BCRP [95]. When administered as a single 100 mg dose on healthy volunteers, venetoclax increased digoxin C_max_ by 35% and AUC_0–∞_ by 9%, suggesting that, in a clinical setting, venetoclax can affect the clearance of digoxin and possibly other P-gp substrates. To minimize potential interactions, digoxin and other narrow therapeutic index P-gp substrates should be administered six hours prior to venetoclax administration [96]. On the other hand, in the same study, it was concluded that venetoclax pharmacokinetics were unchanged by the concurrent administration of digoxin [96]. In another study involving the P-gp substrate azithromycin, only modest changes in venetoclax exposures were observed, indicating that no dose adjustment would be needed [97]. However, the instructions on venetoclax suggest a minimum 50% decrease in venetoclax dose when it is used in combination with P-gp inhibitors [92].

In conclusion, the management of venetoclax-induced neutropenia necessitates careful consideration of drug–drug interactions. This is particularly crucial as antimicrobials, intended to mitigate the heightened infection risk, can contribute to prolonged exposure to venetoclax and its associated toxicity. Therefore, vigilance in assessing and addressing potential interactions is vital to ensure the safe and effective use of venetoclax in clinical practice.

## 7. Conclusions

Venetoclax, a BCL-2 inhibitor with high oral bioavailability, has significantly improved the treatment landscape for leukemia, particularly CLL/SLL and AML, by overcoming anti-apoptotic mechanisms and achieving high remission rates and sustained responses. However, venetoclax-induced neutropenia remains a critical issue that demands vigilant management in clinical settings. This challenge is exacerbated by the immunosuppression inherent to the disease, hematological toxicities from concurrent therapies, and the typically older age and comorbidities of leukemia patients. Additionally, long-term venetoclax treatment may prompt clonal evolution and disease relapse.

Addressing venetoclax-induced neutropenia involves understanding and monitoring patient-specific and disease-specific risk factors, as well as adjusting dosages and treatment schedules to minimize hematological toxicity. A proactive and adaptable treatment strategy, including dose modifications and the management of adverse effects throughout the treatment and intracycle periods, is essential. Prophylactic strategies against infection must also account for potential drug–drug interactions due to venetoclax’s unique pharmacokinetics.

The second major challenge is preventing venetoclax resistance while maintaining its therapeutic effects. Current evidence suggests that limiting the duration of treatment cycles can help preserve drug sensitivity and reduce the risk of severe neutropenia. Research is ongoing to identify strategies to counteract primary and adaptive resistance mechanisms in leukemia, such as combining venetoclax with inhibitors of other anti-apoptotic proteins like MCL-1. This approach may lead to a more effective therapeutic strategy that maximizes efficacy against hematological malignancies through potent, apoptosis-inducing activity while limiting collateral damage to healthy cells.

## Figures and Tables

**Table 1:** Grade III/IV neutropenia in clinical trials of venetoclax, in CLL/SLL.

Study	Citation	Design	Grade III/IV Neutropenia
Phase 1	Roberts et al., 2016 [42]	150–1200 mg	41%
Phase 1b	Seymour et al., 2017; Ma et al., 2021 [44,45]	200–600 mg	53%
Phase 1b	Flinn et al., 2019 [53]	100–400 mg	53–58%
Phase 2	Stilgenbauer et al., 2018 [49]	400 mg	40%
Phase 2	Coutre et al., 2018 [54]	400 mg	50%
Phase 2	Jones et al., 2018 [55]	400 mg	51%
Phase 3 (VENICE II)	Cochrane et al., 2021 [56]	400 mg	32%
Phase 3 (MURANO)	Seymour et al., 2018; Kater et al., 2020 [21,52]	400 mg	58%
Phase 3 (CLL14)	Fisher et al., 2019; Al-Sawaf et al., 2020 [57,58]	400 mg	53%
Phase 3 (CLL13)	Eichhorst et al., 2021 [59]	400 mg	40–45%

**Table 2:** Grade III/IV neutropenia in clinical trials of venetoclax in AML.

Study	Citation	Design	Grade III/IV Neutropenia
Phase 1b	DiNardo et al., 2019 [61]	400–1200 mg	43%
Phase 1b (CAVEAT)	Chua et al., 2020 [66]	50–600 mg	55%
Phase 1b/2	Wei et al., 2019 [62]	600 mg	42%
Phase 2	Konopleva et al., 2016 [60]	1200 mg	31%
Phase 3 (VIALE-A)	DiNardo et al., 2020 [19]	400 mg	42%
Phase 3 (VIALE-C)	Wei et al., 2020–2021 [22,63]	600 mg	32–47%

## Data Availability

Data sharing is not applicable.

## References

[1] Shimada A. (2019). Hematological Malignancies and Molecular Targeting Therapy. Eur. J. Pharmacol..

[2] Rodriguez-Abreu D., Bordoni A., Zucca E. (2007). Epidemiology of Hematological Malignancies. Ann. Oncol. Off. J. Eur. Soc. Med. Oncol..

[3] Siegel R.L., Miller K.D., Fuchs H.E., Jemal A. (2021). Cancer Statistics, 2021. CA A Cancer J. Clin..

[4] Australian Institute of Health and Welfare Cancer Data in Australia, About-Australian Institute of Health and Welfare. https://www.aihw.gov.au/reports/cancer/cancer-data-in-australia/contents/about.

[5] National Cancer Institute Surveillance, Epidemiology and End Results Program. https://seer.cancer.gov/statistics-network/explorer/application.html?site=96&data_type=4&graph_type=2&compareBy=sex&chk_sex_1=1&relative_survival_interval=5&race=1&age_range=1&hdn_stage=101&advopt_precision=1&advopt_show_ci=on&hdn_view=0&advopt_show_apc=on&advopt_display=1#resultsRegion0.

[6] Montserrat E. (2006). New Prognostic Markers in CLL. Hematol. Am. Soc. Hematol. Educ. Program.

[7] Eichhorst B., Hallek M. (2016). Prognostication of Chronic Lymphocytic Leukemia in the Era of New Agents. Hematol. Am. Soc. Hematol. Educ. Program.

[8] Ghia P., Ferreri A.M., Galigaris-Cappio F. (2007). Chronic Lymphocytic Leukemia. Crit. Rev. Oncol. Hematol..

[9] Parikh S.A., Rabe K.G., Kay N.E., Call T.G., Ding W., Schwager S.M., Bowen D.A., Conte M., Jelinek D.F., Slager S.L. (2014). Chronic Lymphocytic Leukemia in Young (≤55 Years) Patients: A Comprehensive Analysis of Prognostic Factors and Outcomes. Haematologica.

[10] De Lima M., O’Brien S., Lerner S., Keating M.J. (1998). Chronic Lymphocytic Leukemia in the Young Patient. Semin. Oncol..

[11] Richardson D.B., Wing S., Schroeder J., Schmitz-Feuerhake I., Hoffmann W. (2005). Ionizing Radiation and Chronic Lymphocytic Leukemia. Environ. Health Perspect..

[12] De Kouchkovsky I., Abdul-Hay M. (2016). Acute Myeloid Leukemia: A Comprehensive Review and 2016 Update. Blood Cancer J..

[13] (2013). Genomic and Epigenomic Landscapes of Adult De Novo Acute Myeloid Leukemia. N. Engl. J. Med..

[14] Gilliland D.G., Griffin J.D. (2002). The Roles of FLT3 in Hematopoiesis and Leukemia. Blood.

[15] Daver N.G., Maiti A., Kadia T.M., Vyas P., Majeti R., Wei A.H., Garcia-Manero G., Craddock C., Sallman D.A., Kantarjian H.M. (2022). TP53-Mutated Myelodysplastic Syndrome and Acute Myeloid Leukemia: Biology, Current Therapy, and Future Directions. Cancer Discov..

[16] Ningombam A., Verma D., Kumar R., Singh J., Ali M.S., Pandey A.K., Singh I., Bakhshi S., Sharma A., Pushpam D. (2023). Prognostic Relevance of NPM1, CEBPA, and FLT3 Mutations in Cytogenetically Normal Adult AML Patients. Am. J. Blood Res..

[17] Gajbhiye S.S., Karwa A.R., Dhok A., Jadhav S.S. (2022). Clinical and Etiological Profiles of Patients With Pancytopenia in a Tertiary Care Hospital. Cureus.

[18] Döhner H., Wei A.H., Appelbaum F.R., Craddock C., DiNardo C.D., Dombret H., Ebert B.L., Fenaux P., Godley L.A., Hasserjian R.P. (2022). Diagnosis and Management of AML in Adults: 2022 Recommendations from an International Expert Panel on Behalf of the ELN. Blood.

[19] DiNardo C.D., Jonas B.A., Pullarkat V., Thirman M.J., Garcia J.S., Wei A.H., Konopleva M., Döhner H., Letai A., Fenaux P. (2020). Azacitidine and Venetoclax in Previously Untreated Acute Myeloid Leukemia. N. Engl. J. Med..

[20] Fischer K., Al-Sawaf O., Bahlo J., Fink A.-M., Tandon M., Dixon M., Robrecht S., Warburton S., Humphrey K., Samoylova O. (2019). Venetoclax and Obinutuzumab in Patients with CLL and Coexisting Conditions. N. Engl. J. Med..

[21] Seymour J.F., Kipps T.J., Eichhorst B., Hillmen P., D’Rozario J., Assouline S., Owen C., Gerecitano J., Robak T., De la Serna J. (2018). Venetoclax-Rituximab in Relapsed or Refractory Chronic Lymphocytic Leukemia. N. Engl. J. Med..

[22] Wei A.H., Montesinos P., Ivanov V., DiNardo C.D., Novak J., Laribi K., Kim I., Stevens D.A., Fiedler W., Pagoni M. (2020). Venetoclax plus LDAC for Newly Diagnosed AML Ineligible for Intensive Chemotherapy: A Phase 3 Randomized Placebo-Controlled Trial. Blood.

[23] Siddiqui M., Konopleva M. (2021). Keeping up with Venetoclax for Leukemic Malignancies: Key Findings, Optimal Regimens, and Clinical Considerations. Expert Rev. Clin. Pharmacol..

[24] Elhadi M., Khaled A., Msherghi A. (2021). Infectious Diseases as a Cause of Death among Cancer Patients: A Trend Analysis and Population-Based Study of Outcome in the United States Based on the Surveillance, Epidemiology, and End Results Database. Infect. Agent. Cancer.

[25] On S., Rath C.G., Lan M., Wu B., Lau K.M., Cheung E., Alegria W., Young R., Tan M., Kim C. (2022). Characterisation of Infections in Patients with Acute Myeloid Leukaemia Receiving Venetoclax and a Hypomethylating Agent. Br. J. Haematol..

[26] Vaux D.L., Cory S., Adams J.M. (1988). Bcl-2 Gene Promotes Haemopoietic Cell Survival and Cooperates with c-Myc to Immortalize Pre-B Cells. Nature.

[27] Adams J.M., Cory S. (1998). The Bcl-2 Protein Family: Arbiters of Cell Survival. Science.

[28] Chen L., Willis S.N., Wei A., Smith B.J., Fletcher J.I., Hinds M.G., Colman P.M., Day C.L., Adams J.M., Huang D.C.S. (2005). Differential Targeting of Prosurvival Bcl-2 Proteins by Their BH3-Only Ligands Allows Complementary Apoptotic Function. Mol. Cell.

[29] Willis S.N., Chen L., Dewson G., Wei A., Naik E., Fletcher J.I., Adams J.M., Huang D.C.S. (2005). Proapoptotic Bak Is Sequestered by Mcl-1 and Bcl-xL, but Not Bcl-2, until Displaced by BH3-Only Proteins. Genes. Dev..

[30] Czabotar P.E., Lessene G., Strasser A., Adams J.M. (2014). Control of Apoptosis by the BCL-2 Protein Family: Implications for Physiology and Therapy. Nat. Rev. Mol. Cell Biol..

[31] Chittenden T. (2002). BH3 Domains: Intracellular Death-Ligands Critical for Initiating Apoptosis. Cancer Cell.

[32] Qian S., Wei Z., Yang W., Huang J., Yang Y., Wang J. (2022). The Role of BCL-2 Family Proteins in Regulating Apoptosis and Cancer Therapy. Front. Oncol..

[33] Ashkenazi A., Fairbrother W.J., Leverson J.D., Souers A.J. (2017). From Basic Apoptosis Discoveries to Advanced Selective BCL-2 Family Inhibitors. Nat. Rev. Drug Discov..

[34] Lampson B.L., Davids M.S. (2017). The Development and Current Use of BCL-2 Inhibitors for the Treatment of Chronic Lymphocytic Leukemia. Curr. Hematol. Malig. Rep..

[35] O’Brien S.M., Cunningham C.C., Golenkov A.K., Turkina A.G., Novick S.C., Rai K.R. (2005). Phase I to II Multicenter Study of Oblimersen Sodium, a Bcl-2 Antisense Oligonucleotide, in Patients with Advanced Chronic Lymphocytic Leukemia. J. Clin. Oncol..

[36] O’Brien S.M., Claxton D.F., Crump M., Faderl S., Kipps T., Keating M.J., Viallet J., Cheson B.D. (2009). Phase I Study of Obatoclax Mesylate (GX15-070), a Small Molecule Pan-Bcl-2 Family Antagonist, in Patients with Advanced Chronic Lymphocytic Leukemia. Blood.

[37] Roberts A.W., Seymour J.F., Brown J.R., Wierda W.G., Kipps T.J., Khaw S.L., Carney D.A., He S.Z., Huang D.C.S., Xiong H. (2012). Substantial Susceptibility of Chronic Lymphocytic Leukemia to BCL2 Inhibition: Results of a Phase I Study of Navitoclax in Patients with Relapsed or Refractory Disease. J. Clin. Oncol..

[38] Zhang H., Nimmer P.M., Tahir S.K., Chen J., Fryer R.M., Hahn K.R., Iciek L.A., Morgan S.J., Nasarre M.C., Nelson R. (2007). Bcl-2 Family Proteins Are Essential for Platelet Survival. Cell Death Differ..

[39] Center for Drug Evaluation and Research FDA Grants Regular Approval to Venetoclax in Combination for Untreated Acute Myeloid Leukemia. https://www.fda.gov/drugs/resources-information-approved-drugs/fda-grants-regular-approval-venetoclax-combination-untreated-acute-myeloid-leukemia.

[40] Center for Drug Evaluation and Research FDA Approves Venetoclax for CLL and SLL. https://www.fda.gov/drugs/resources-information-approved-drugs/fda-approves-venetoclax-cll-and-sll.

[41] Souers A.J., Leverson J.D., Boghaert E.R., Ackler S.L., Catron N.D., Chen J., Dayton B.D., Ding H., Enschede S.H., Fairbrother W.J. (2013). ABT-199, a Potent and Selective BCL-2 Inhibitor, Achieves Antitumor Activity While Sparing Platelets. Nat. Med..

[42] Roberts A.W., Davids M.S., Pagel J.M., Kahl B.S., Puvvada S.D., Gerecitano J.F., Kipps T.J., Anderson M.A., Brown J.R., Gressick L. (2016). Targeting BCL2 with Venetoclax in Relapsed Chronic Lymphocytic Leukemia. N. Engl. J. Med..

[43] Roberts A.W., Advani R.H., Kahl B.S., Persky D., Sweetenham J.W., Carney D.A., Yang J., Busman T.B., Enschede S.H., Humerickhouse R.A. (2015). Phase 1 Study of the Safety, Pharmacokinetics, and Antitumour Activity of the BCL2 Inhibitor Navitoclax in Combination with Rituximab in Patients with Relapsed or Refractory CD20+ Lymphoid Malignancies. Br. J. Haematol..

[44] Seymour J.F., Ma S., Brander D.M., Choi M.Y., Barrientos J., Davids M.S., Anderson M.A., Beaven A.W., Rosen S.T., Tam C.S. (2017). Venetoclax plus Rituximab in Relapsed or Refractory Chronic Lymphocytic Leukaemia: A Phase 1b Study. Lancet Oncol..

[45] Ma S., Seymour J.F., Brander D.M., Kipps T.J., Choi M.Y., Anderson M.A., Humphrey K., Al Masud A., Pesko J., Nandam R. (2021). Efficacy of Venetoclax plus Rituximab for Relapsed CLL: 5-Year Follow-up of Continuous or Limited- Duration Therapy. Blood.

[46] Delgado J., Espinet B., Oliveira A.C., Abrisqueta P., de la Serna J., Collado R., Loscertales J., Lopez M., Hernandez-Rivas J.A., Ferra C. (2012). Chronic Lymphocytic Leukaemia with 17p Deletion: A Retrospective Analysis of Prognostic Factors and Therapy Results. Br. J. Haematol..

[47] Maddocks K.J., Ruppert A.S., Lozanski G., Heerema N.A., Zhao W., Abruzzo L., Lozanski A., Davis M., Gordon A., Smith L.L. (2015). Etiology of Ibrutinib Therapy Discontinuation and Outcomes in Patients with Chronic Lymphocytic Leukemia. JAMA Oncol..

[48] Furman R.R., Sharman J.P., Coutre S.E., Cheson B.D., Pagel J.M., Hillmen P., Barrientos J.C., Zelenetz A.D., Kipps T.J., Flinn I. (2014). Idelalisib and Rituximab in Relapsed Chronic Lymphocytic Leukemia. N. Engl. J. Med..

[49] Stilgenbauer S., Eichhorst B., Schetelig J., Hillmen P., Seymour J.F., Coutre S., Jurczak W., Mulligan S.P., Schuh A., Assouline S. (2018). Venetoclax for Patients With Chronic Lymphocytic Leukemia With 17p Deletion: Results From the Full Population of a Phase II Pivotal Trial. J. Clin. Oncol..

[50] Stilgenbauer S., Eichhorst B., Schetelig J., Coutre S., Seymour J.F., Munir T., Puvvada S.D., Wendtner C.-M., Roberts A.W., Jurczak W. (2016). Venetoclax in Relapsed or Refractory Chronic Lymphocytic Leukaemia with 17p Deletion: A Multicentre, Open-Label, Phase 2 Study. Lancet Oncol..

[51] Kater A.P., Seymour J.F., Hillmen P., Eichhorst B., Langerak A.W., Owen C., Verdugo M., Wu J., Punnoose E.A., Jiang Y. (2019). Fixed Duration of Venetoclax-Rituximab in Relapsed/Refractory Chronic Lymphocytic Leukemia Eradicates Minimal Residual Disease and Prolongs Survival: Post-Treatment Follow-Up of the MURANO Phase III Study. J. Clin. Oncol..

[52] Kater A.P., Wu J.Q., Kipps T., Eichhorst B., Hillmen P., D’Rozario J., Assouline S., Owen C., Robak T., de la Serna J. (2020). Venetoclax Plus Rituximab in Relapsed Chronic Lymphocytic Leukemia: 4-Year Results and Evaluation of Impact of Genomic Complexity and Gene Mutations From the MURANO Phase III Study. J. Clin. Oncol..

[53] Flinn I.W., Gribben J.G., Dyer M.J.S., Wierda W., Maris M.B., Furman R.R., Hillmen P., Rogers K.A., Iyer S.P., Quillet-Mary A. (2019). Phase 1b Study of Venetoclax-Obinutuzumab in Previously Untreated and Relapsed/Refractory Chronic Lymphocytic Leukemia. Blood.

[54] Coutre S., Choi M., Furman R.R., Eradat H., Heffner L., Jones J.A., Chyla B., Zhou L., Agarwal S., Waskiewicz T. (2018). Venetoclax for Patients with Chronic Lymphocytic Leukemia Who Progressed during or after Idelalisib Therapy. Blood.

[55] Jones J.A., Mato A.R., Wierda W.G., Davids M.S., Choi M., Cheson B.D., Furman R.R., Lamanna N., Barr P.M., Zhou L. (2018). Venetoclax for Chronic Lymphocytic Leukaemia Progressing after Ibrutinib: An Interim Analysis of a Multicentre, Open-Label, Phase 2 Trial. Lancet Oncol..

[56] Cochrane T., Enrico A., Gomez-Almaguer D., Hadjiev E., Lech-Maranda E., Masszi T., Nikitin E., Robak T., Weinkove R., Wu S.-J. (2022). Impact of Venetoclax Monotherapy on the Quality of Life of Patients with Relapsed or Refractory Chronic Lymphocytic Leukemia: Results from the Phase 3b VENICE II Trial. Leuk. Lymphoma.

[57] Fischer K., Ritgen M., Al-Sawaf O., Robrecht S., Tandon M., Fink A.-M., Schilhabel A., Stübig T., Brüggemann M., Jiang Y. (2019). Quantitative Analysis of Minimal Residual Disease (MRD) Shows High Rates of Undetectable MRD after Fixed-Duration Chemotherapy-Free Treatment and Serves As Surrogate Marker for Progression-Free Survival: A Prospective Analysis of the Randomized CLL14 Trial. Blood.

[58] Al-Sawaf O., Zhang C., Tandon M., Sinha A., Fink A.-M., Robrecht S., Samoylova O., Liberati A.M., Pinilla-Ibarz J., Opat S. (2020). Venetoclax plus Obinutuzumab versus Chlorambucil plus Obinutuzumab for Previously Untreated Chronic Lymphocytic Leukaemia (CLL14): Follow-up Results from a Multicentre, Open-Label, Randomised, Phase 3 Trial. Lancet Oncol..

[59] Eichhorst B., Niemann C.U., Kater A.P., Fürstenau M., von Tresckow J., Zhang C., Robrecht S., Gregor M., Juliusson G., Thornton P. (2023). First-Line Venetoclax Combinations in Chronic Lymphocytic Leukemia. N. Engl. J. Med..

[60] Konopleva M., Pollyea D.A., Potluri J., Chyla B., Hogdal L., Busman T., McKeegan E., Salem A.H., Zhu M., Ricker J.L. (2016). Efficacy and Biological Correlates of Response in a Phase II Study of Venetoclax Monotherapy in Patients with Acute Myelogenous Leukemia. Cancer Discov..

[61] DiNardo C.D., Pratz K., Pullarkat V., Jonas B.A., Arellano M., Becker P.S., Frankfurt O., Konopleva M., Wei A.H., Kantarjian H.M. (2019). Venetoclax Combined with Decitabine or Azacitidine in Treatment-Naive, Elderly Patients with Acute Myeloid Leukemia. Blood.

[62] Wei A.H., Strickland S.A., Hou J.-Z., Fiedler W., Lin T.L., Walter R.B., Enjeti A., Tiong I.S., Savona M., Lee S. (2019). Venetoclax Combined With Low-Dose Cytarabine for Previously Untreated Patients With Acute Myeloid Leukemia: Results From a Phase Ib/II Study. J. Clin. Oncol..

[63] Wei A.H., Panayiotidis P., Montesinos P., Laribi K., Ivanov V., Kim I., Novak J., Stevens D.A., Fiedler W., Pagoni M. (2021). 6-Month Follow-up of VIALE-C Demonstrates Improved and Durable Efficacy in Patients with Untreated AML Ineligible for Intensive Chemotherapy (141/150). Blood Cancer J..

[64] de Leeuw D.C., Ossenkoppele G.J., Janssen J.J.W.M. (2022). Older Patients with Acute Myeloid Leukemia Deserve Individualized Treatment. Curr. Oncol. Rep..

[65] Lucijanic M., Tomasovic-Loncaric C., Stoos-Veic T., De Both T., Jalsenjak B., Kusec R. (2024). Myeloid Sarcoma of the Urinary Bladder as the Presenting Feature of Secondary Acute Myeloid Leukemia, Successfully Treated with Venetoclax and Azacitidine. Ann. Hematol..

[66] Chua C.C., Roberts A.W., Reynolds J., Fong C.Y., Ting S.B., Salmon J.M., MacRaild S., Ivey A., Tiong I.S., Fleming S. (2020). Chemotherapy and Venetoclax in Elderly Acute Myeloid Leukemia Trial (CAVEAT): A Phase Ib Dose-Escalation Study of Venetoclax Combined With Modified Intensive Chemotherapy. J. Clin. Oncol..

[67] Waggoner M., Katsetos P.-C.J., Thomas M.E., Galinsky B.I., Fox P.-C.H. (2022). Practical Management of the Venetoclax-Treated Patient in Chronic Lymphocytic Leukemia and Acute Myeloid Leukemia. J. Adv. Pract. Oncol..

[68] Ma R.-M., Chen C.-Z., Zhang W., You J., Huang D.-P., Guo G.-L. (2016). Prognostic Value of Chemotherapy-Induced Neutropenia at the First Cycle in Invasive Breast Cancer. Medicine.

[69] Lalami Y., Klastersky J. (2017). Impact of Chemotherapy-Induced Neutropenia (CIN) and Febrile Neutropenia (FN) on Cancer Treatment Outcomes: An Overview about Well-Established and Recently Emerging Clinical Data. Crit. Rev. Oncol. Hematol..

[70] Leverson J.D., Phillips D.C., Mitten M.J., Boghaert E.R., Diaz D., Tahir S.K., Belmont L.D., Nimmer P., Xiao Y., Ma X.M. (2015). Exploiting Selective BCL-2 Family Inhibitors to Dissect Cell Survival Dependencies and Define Improved Strategies for Cancer Therapy. Sci. Transl. Med..

[71] Samuels C., Abbott D., Niemiec S., Tobin J., Falco A., Halsema K., Kamdar M. (2022). Evaluation and Associated Risk Factors for Neutropenia with Venetoclax and Obinutuzumab in the Treatment of Chronic Lymphocytic Leukemia. Cancer Rep..

[72] Pratz K.W., DiNardo C.D., Selleslag D., Li J., Yamamoto K., Konopleva M., Stevens D., Kantarjian H., Traina F., Venditti A. (2022). Postremission Cytopenia Management in Patients with Acute Myeloid Leukemia Treated with Venetoclax and Azacitidine in VIALE-A. Am. J. Hematol..

[73] Prosty C., Katergi K., Nguyen A., Luo O.D., Sorin M., Cherniak V., Sebag M., Demir K., McDonald E.G., Lee T.C. (2023). Infectious Complications of Venetoclax Treatment of Hematologic Malignancies: A Systematic Review and Meta-Analysis. Blood Adv..

[74] Aiba M., Shigematsu A., Suzuki T., Miyagishima T. (2023). Shorter Duration of Venetoclax Administration to 14 Days Has Same Efficacy and Better Safety Profile in Treatment of Acute Myeloid Leukemia. Ann. Hematol..

[75] Samra B., Konopleva M., Isidori A., Daver N., DiNardo C. (2020). Venetoclax-Based Combinations in Acute Myeloid Leukemia: Current Evidence and Future Directions. Front. Oncol..

[76] Frustaci A.M., Del Poeta G., Visentin A., Sportoletti P., Fresa A., Vitale C., Murru R., Chiarenza A., Sanna A., Mauro F.R. (2022). Coexisting Conditions and Concomitant Medications Do Not Affect Venetoclax Management and Survival in Chronic Lymphocytic Leukemia. Ther. Adv. Hematol..

[77] Ong F., Kim K., Konopleva M.Y. (2022). Venetoclax Resistance: Mechanistic Insights and Future Strategies. Cancer Drug Resist..

[78] DiNardo C.D., Tiong I.S., Quaglieri A., MacRaild S., Loghavi S., Brown F.C., Thijssen R., Pomilio G., Ivey A., Salmon J.M. (2020). Molecular Patterns of Response and Treatment Failure after Frontline Venetoclax Combinations in Older Patients with AML. Blood.

[79] Blombery P., Anderson M.A., Gong J.-N., Thijssen R., Birkinshaw R.W., Thompson E.R., Teh C.E., Nguyen T., Xu Z., Flensburg C. (2019). Acquisition of the Recurrent Gly101Val Mutation in BCL2 Confers Resistance to Venetoclax in Patients with Progressive Chronic Lymphocytic Leukemia. Cancer Discov..

[80] Liu J., Chen Y., Yu L., Yang L. (2022). Mechanisms of Venetoclax Resistance and Solutions. Front. Oncol..

[81] Portell C.A., Jegede O., Wagner-Johnston N.D., Nowakowski G.S., Fletcher C.D., Cohen J.B., David K.A., Khan N., Rosenstein L.J., Kahl B.S. (2021). Phase II Study of Venetoclax in Combination with Obinutuzumab and Bendamustine in Patients with High Tumor Burden Follicular Lymphoma As Front Line Therapy (PrECOG 0403). Blood.

[82] Kumar S.K., Harrison S.J., Cavo M., de la Rubia J., Popat R., Gasparetto C., Hungria V., Salwender H., Suzuki K., Kim I. (2020). Venetoclax or Placebo in Combination with Bortezomib and Dexamethasone in Patients with Relapsed or Refractory Multiple Myeloma (BELLINI): A Randomised, Double-Blind, Multicentre, Phase 3 Trial. Lancet Oncol..

[83] Savvides P., Terrin N., Erban J., Selker H.P. (2003). Development and Validation of a Patient-Specific Predictive Instrument for the Need for Dose Reduction in Chemotherapy for Breast Cancer: A Potential Decision Aid for the Use of Myeloid Growth Factors. Support. Care Cancer.

[84] Buckley S.A., Othus M., Vainstein V., Abkowitz J.L., Estey E.H., Walter R.B. (2014). Prediction of Adverse Events during Intensive Induction Chemotherapy for Acute Myeloid Leukemia or High-Grade Myelodysplastic Syndromes. Am. J. Hematol..

[85] DiNardo C.D., Lachowiez C.A., Takahashi K., Loghavi S., Kadia T., Daver N., Xiao L., Adeoti M., Short N.J., Sasaki K. (2022). Venetoclax combined with FLAG-IDA induction and consolidation in newly diagnosed acute myeloid leukemia. Am. J. Hematol..

[86] Maiti A., Konopleva M.Y. (2022). How We Incorporate Venetoclax in Treatment Regimens for Acute Myeloid Leukemia. Cancer J..

[87] Kala S.G., Chinni S. (2021). Development and Characterization of Venetoclax Nanocrystals for Oral Bioavailability Enhancement. AAPS PharmSciTech.

[88] Salem A.H., Agarwal S.K., Dunbar M., Enschede S.L.H., Humerickhouse R.A., Wong S.L. (2017). Pharmacokinetics of Venetoclax, a Novel BCL-2 Inhibitor, in Patients With Relapsed or Refractory Chronic Lymphocytic Leukemia or Non-Hodgkin Lymphoma. J. Clin. Pharmacol..

[89] Tariq S., Tariq S., Khan M., Azhar A., Baig M. (2020). Venetoclax in the Treatment of Chronic Lymphocytic Leukemia: Evidence, Expectations, and Future Prospects. Cureus.

[90] Jones A.K., Freise K.J., Agarwal S.K., Humerickhouse R.A., Wong S.L., Salem A.H. (2016). Clinical Predictors of Venetoclax Pharmacokinetics in Chronic Lymphocytic Leukemia and Non-Hodgkin’s Lymphoma Patients: A Pooled Population Pharmacokinetic Analysis. AAPS J..

[91] Agarwal S.K., DiNardo C.D., Potluri J., Dunbar M., Kantarjian H.M., Humerickhouse R.A., Wong S.L., Menon R.M., Konopleva M.Y., Salem A.H. (2017). Management of Venetoclax-Posaconazole Interaction in Acute Myeloid Leukemia Patients: Evaluation of Dose Adjustments. Clin. Ther..

[92] Venetoclax FDA Prescribing Information. https://www.accessdata.fda.gov/drugsatfda_docs/label/2019/208573s013lbl.pdf.

[93] Freise K.J., Shebley M., Salem A.H. (2017). Quantitative Prediction of the Effect of CYP3A Inhibitors and Inducers on Venetoclax Pharmacokinetics Using a Physiologically Based Pharmacokinetic Model. J. Clin. Pharmacol..

[94] Richard-Carpentier G., DiNardo C.D. (2019). Venetoclax for the Treatment of Newly Diagnosed Acute Myeloid Leukemia in Patients Who Are Ineligible for Intensive Chemotherapy. Ther. Adv. Hematol..

[95] Fan W., Guo J., Zhang Y., Zhang R., Lin B. (2024). Venetoclax Dose Adjustment Due to Drug-Drug Interactions: A Case Report and Literature Review. Anticancer. Drugs.

[96] Chiney M.S., Menon R.M., Bueno O.F., Tong B., Salem A.H. (2018). Clinical Evaluation of P-Glycoprotein Inhibition by Venetoclax: A Drug Interaction Study with Digoxin. Xenobiotica.

[97] Agarwal S.K., Tong B., Bueno O.F., Menon R.M., Salem A.H. (2018). Effect of Azithromycin on Venetoclax Pharmacokinetics in Healthy Volunteers: Implications for Dosing Venetoclax with P-Gp Inhibitors. Adv. Ther..

